# Supplementary figures and images for: MFI-Net: multi-level feature invertible network image concealment technique
Source: PeerJ Comput Sci. 2025 Feb 14;11:e2668. doi: 10.7717/peerj-cs.2668 (PMC11888862; doi:10.7717/peerj-cs.2668)

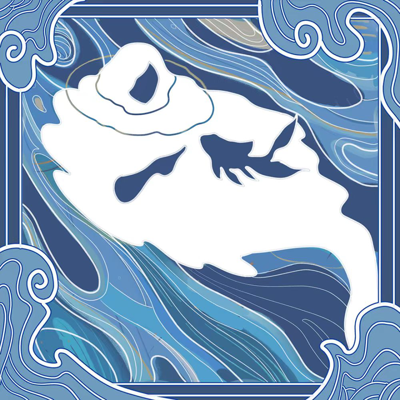

Supplement: Supplemental Information 2 — The digital artwork image was purchased and used for practical application testing. [file peerj-cs-11-2668-s002.png]

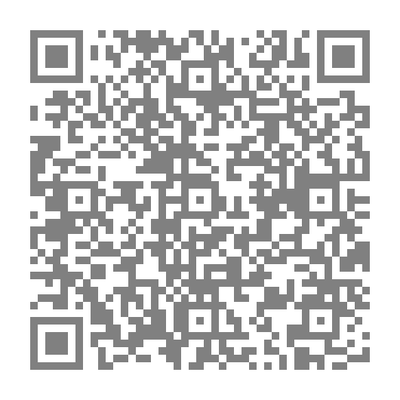

Supplement: Supplemental Information 3 — The image was used for practical application testing, generated by a Python library. [file peerj-cs-11-2668-s003.png]
